# Kaolin Foliar Application Has a Stimulatory Effect on Phenylpropanoid and Flavonoid Pathways in Grape Berries

**DOI:** 10.3389/fpls.2016.01150

**Published:** 2016-08-08

**Authors:** Artur Conde, Diana Pimentel, Andreia Neves, Lia-Tânia Dinis, Sara Bernardo, Carlos M. Correia, Hernâni Gerós, José Moutinho-Pereira

**Affiliations:** ^1^Centre for the Research and Technology of Agro-Environmental and Biological Sciences, University of Trás-os-Montes e Alto DouroVila Real, Portugal; ^2^Grupo de Investigação em Biologia Vegetal Aplicada e Inovação Agroalimentar (AgroBioPlant), Departamento de Biologia, Universidade do MinhoBraga, Portugal; ^3^Department of Biology, Centre of Molecular and Environmental Biology, University of MinhoBraga, Portugal

**Keywords:** grape berry, phenylpropanoids, flavonoids, secondary metabolites, metabolic changes, fruit quality, kaolin, stress mitigation

## Abstract

Drought, elevated air temperature, and high evaporative demand are increasingly frequent during summer in grape growing areas like the Mediterranean basin, limiting grapevine productivity and berry quality. The foliar exogenous application of kaolin, a radiation-reflecting inert mineral, has proven effective in mitigating the negative impacts of these abiotic stresses in grapevine and other fruit crops, however, little is known about its influence on the composition of the grape berry and on key molecular mechanisms and metabolic pathways notably important for grape berry quality parameters. Here, we performed a thorough molecular and biochemical analysis to assess how foliar application of kaolin influences major secondary metabolism pathways associated with berry quality-traits, leading to biosynthesis of phenolics and anthocyanins, with a focus on the phenylpropanoid, flavonoid (both flavonol- and anthocyanin-biosynthetic) and stilbenoid pathways. In grape berries from different ripening stages, targeted transcriptional analysis by qPCR revealed that several genes involved in these pathways—*VvPAL1, VvC4H1, VvSTSs, VvCHS1, VvFLS1, VvDFR*, and *VvUFGT*—were more expressed in response to the foliar kaolin treatment, particularly in the latter maturation phases. In agreement, enzymatic activities of phenylalanine ammonia lyase (PAL), flavonol synthase (FLS), and UDP-glucose:flavonoid 3-O-glucosyltransferase (UFGT) were about two-fold higher in mature or fully mature berries from kaolin-treated plants, suggesting regulation also at a transcriptional level. The expression of the glutathione S-transferase *VvGST4*, and of the tonoplast anthocyanin transporters *VvMATE1* and *VvABCC1* were also all significantly increased at véraison and in mature berries, thus, when anthocyanins start to accumulate in the vacuole, in agreement with previously observed higher total concentrations of phenolics and anthocyanins in berries from kaolin-treated plants, especially at full maturity stage. Metabolomic analysis by reverse phase LC-QTOF-MS confirmed several kaolin-induced modifications including a significant increase in the quantities of several secondary metabolites including flavonoids and anthocyanins in the latter ripening stages, probably resulting from the general stimulation of the phenylpropanoid and flavonoid pathways.

## Introduction

Grapevine (*Vitis vinifera* L.) is a perennial woody plant with a great impact in the global economy, abundantly cultivated in areas with Mediterranean climates and spreading across temperate to semi-dry areas. Abiotic conditions, such as soil and atmospheric humidity, intense drought, and temperatures, have high impact on grape yield and wine quality (Chaves et al., 2010; Lovisolo et al., 2010). In Mediterranean areas, extended summer droughts and higher temperatures are increasingly expected (Fraga et al., 2012; Hannah et al., 2013) and climate change is undoubtedly having a negative impact in viticulture, including changes in grape-growing geographical area, therefore the development and application of stress mitigation strategies and of more sustainable agricultural practices is of utmost importance for grape production and winemaking industry.

In this context, the application of exogenous compounds that could maintain or even improve plant productivity or fruit quality under such environmental stresses are beginning to be experimented but, despite promising results yielded in some crops (Hose et al., 2000; Li et al., 2004; Seckin et al., 2009; Du et al., 2013; Zhou et al., 2014), in grapevine these strategies have so far been less explored. Kaolin, Al_2_Si_2_O_5_(OH)_4_, is an inert clay mineral that reflects potentially damaging ultraviolet and infrared radiation and transmits photosynthetically active radiation, resulting in leaf temperature decrease and photosynthetic efficiency increase (Glenn and Puterka, 2005). Its exogenous application in leaves resulted in positive responses to abiotic stresses in apple, pomegranate and even olive tree (Glenn et al., 2001; Melgarejo et al., 2004; Khaleghi et al., 2015). In grapevines kaolin particle film induced cooler canopy temperatures, lower rates of stomatal conductance under non-limiting soil moisture conditions, protection of photosystem II structure and function in leaves exposed to heat and high solar radiation, and altered total soluble solids content and total anthocyanin amounts (Shellie and Glenn, 2008; Glenn et al., 2010; Ou et al., 2010; Song et al., 2012; Shellie, 2015; Dinis et al., 2016a,b). We recently observed that lower ROS quantities, increased hydroxyl radical scavenging and production of antioxidant compounds, including phenolics, apparently contributing to the protective effect of kaolin in grapevine (Dinis et al., 2016a), but little is known regarding the molecular mechanisms underlying these changes.

Secondary metabolites are indeed extremely important for fruit quality-traits and wine production, namely phenolics, since they contribute to color, flavor, aroma, texture, astringency, and stabilization of wine, and also exhibit antioxidant properties (reviewed by Teixeira et al., 2013). Phenolic compounds are divided in two major groups, nonflavonoid phenolics, and flavonoids (reviewed by Teixeira et al., 2013). Non-flavonoid phenolics comprise hydroxybenzoic acids, hydroxycinnamic acids, volatile phenolics and stilbenes, while flavonoids are C6-C3-C6 polyphenolic compounds and divided into flavonols, flavan-3-ols (catechins/epicatechins, proanthocyanidins, or condensed tannins) and anthocyanins (Kennedy et al., 2000; Verries et al., 2008). Grapevine anthocyanins are anthocyanidins glycosylated or acylglycosylated at the 3′ position of the B ring, thus, flavonoid-3-*O*-monoglycosides, and are responsible for the pigmentation of colored grape berries, from red through blue, hence for wine color (Castellarin et al., 2012). Two major secondary metabolic biochemical pathways underlie the synthesis of a wide range of important phenolic and flavonoid compounds, including anthocyanins: the phenylpropanoid pathway, with the enzyme phenylalanine ammonia lyase (PAL) playing a major role, and the flavonoid pathway, with several important enzymes involved in the formation of the different classes of flavonoids, discussed further ahead. Anthocyanins are stored in the vacuole after being transported across the tonoplast by primary or secondary transporters such as ATP-binding cassette (ABC) transporters (Francisco et al., 2013), as is the case of VvABCC1, dependent on the presence of reduced glutathione (GSH); or multidrug and toxic extrusion (MATE, or anthoMATE) transporters like MATE1 (or AM1) that use the H^+^ gradient to transport mostly acylated anthocyanins (Gomez et al., 2009, 2011), respectively. Glutathione S-transferases (GSTs), with VvGST4 as a paradigmatic case, are very important in anthocyanin stabilization and transport to the vacuole via a non-covalent (ligandin) activity, and a correlation between anthocyanin accumulation and *VvGST* expression profile during berry ripening has already been established (Conn et al., 2008).

Environmental conditions have a strong influence on the secondary metabolism of grape berry cells (Teixeira et al., 2013), that is reflected in grape berry quality. High temperatures decreases anthocyanin biosynthesis and content (Spayd et al., 2002; Mori et al., 2007; Azuma et al., 2012; Carbonell-Bejerano et al., 2013). Genes encoding enzymes involved in flavonoid biosynthesis, as well as regulatory genes and UFGT enzymatic activity are differently affected by heat stress depending on the cultivar and whether these high temperatures are diurnal or nocturnal (Mori et al., 2005, 2007). Exposure to light, however, appears to promote a increase in phenolic, mostly flavonols, and, in many cases but not all, anthocyanin synthesis and content (Spayd et al., 2002; Fujita et al., 2006; Czemmel et al., 2009; Matus et al., 2009; Azuma et al., 2012), but these responses have recently been proposed to be more complex (reviewed by Pillet et al., 2015). Mild water deficit can enhance anthocyanin and stilbenoid synthesis (Mattivi et al., 2006; Castellarin et al., 2007b; Deluc et al., 2011), however flavonol content is either unaltered or decreased (Deluc et al., 2009; Zarrouk et al., 2012). In fact, fruits from grapevines under severe water deficit stress can have lower synthesis and accumulation of phenolics, including anthocyanins, as often this stress is associated with superimposed very high temperatures in the vineyard edaphoclimate.

This work consisted of a thorough molecular and biochemical analysis with the objective of assessing the influence of a foliar application of kaolin on grape berry secondary metabolism. Transcriptional analyses by qPCR, as well as biochemical analyses including enzyme activity measurements, were performed on key metabolic pathways/molecular mechanisms involved in the biosynthesis of phenolics and anthocyanins, with a focus on phenylpropanoid, flavonoid (both flavonol- and anthocyanin-biosynthetic) and stilbenoid pathways. Metabolomic analysis by reverse phase LC-QTOF-MS was also performed to unveil kaolin-induced modifications on several important secondary metabolites in the latter ripening stages.

## Materials and methods

### Grapevine field conditions and sampling

Whole grape berry samples were collected from field-grown “Touriga Nacional” cultivar grapevines (*Vitis vinifera* L.) grafted onto 110-R from the commercial vineyard “Quinta do Vallado,” in the Douro Demarcated Region (Denomination of Origin Douro/Porto), located at Peso da Régua, Portugal (41°09′44.5″N 07°45′58.2″W). The climate is typically Mediterranean-like, with a warm-temperate climate and dry and hot summers, with higher precipitation during winter but very low during the summer (Kottek et al., 2006). Vines were managed without irrigation and grown using standard cultural practices as applied by commercial farmers. Vineyard rows were located on a steep hill with an N-S orientation. Monthly maximum temperature (T_max_) and precipitation values (April to October) were reported in Dinis et al. (2016a).

Three vineyard rows, with 20 plants each, were sprayed in 17th July 2014, at the late green-phase and right before *véraison*, with 5% (w/v) Kaolin (Surround WP; Engelhard Corp., Iselin, NJ), according to previous work done by our team (Dinis et al., 2016a). A second application in the same day was done to ensure Kaolin adhesion uniformity. Other three vineyard lines, with 20 plants each, were maintained as control, i.e., without Kaolin application. All rows are located side-by-side (ensuring the same edaphoclimatic conditions) on a steep hill with an N-S orientation. The vines were 7 years-old, were trained to unilateral cordon and the spurs were pruned to two nodes each with 10–12 nodes per vine.

Grape berry samples treated with kaolin and without treatment, i.e., control, were randomly collected from different positions in the clusters of different plants from different rows in the vine at four ripening stages: on 23th July (late green phase), 21st August (*véraison*, ca. half of the berries per cluster colored), 3rd September (mature), and on 12th September (full mature); and immediately frozen in liquid nitrogen. In all ripening stages, sampling was performed in sunny and relatively hot days, so with relatively similar environmental conditions in all sampling dates. In the sampling procedure, 50–60 berries (about 5 per cluster) were collected always at the same time of the day, at 6.30 p.m. Phenological parameters of control vs. kaolin-treated fully-mature berries, respectively, were as follows: average berry weight—1.89 vs. 1.88 g, pH—3.98 vs. 3.94; total sugars—198.6 vs. 203.6 g/L. No apparent differences in skin to pulp ratio were noticed between control and kaolin-treated berries. The average water contents of control vs. kaolin-treated berries were as follows: 93.1 vs. 94.1% at green stage, 80.9 vs. 80.7% at veraison, 77.0 vs. 75.9% at mature stage, 74.5 vs. 74.4% at full maturation. For véraison sampling, a representative mix of colored and non-colored berries was obtained. This precaution procedure was adopted both in the cluster and for different clusters of the plant, with half of colored and half of non-colored berries collected from each condition and used for each experiment. The véraison rate was apparently similar between conditions with no apparent phonological displacement. No difference on véraison proportion between treatments was observable.

All berries selected for sampling were totally clean and without any trace or residue of kaolin. Berries were deseeded and ground to a fine powder under liquid nitrogen refrigeration and stored in −80°C. For RNA extraction, metabolite extraction and enzymatic activity assays, 6–8 randomly collected berries were used for grinding and sample homogenization.

### Metabolomic analysis by reverse phase LC-QTOF-MS

Reverse phase LC-QTOF-MS analysis was used to analyze how foliar kaolin application influenced grape berry secondary metabolome. Secondary metabolites were extracted from lyophilized powdered grape berry samples with 50% ethanol. After concentration in vacuum for ethanol removal, the extract was re-suspended with water. The aqueous solution was subsequently extracted with light petroleum and ethyl acetate, respectively. Samples were then evaporated under reduced pressure. Metabolite profiling analyses were performed with a liquid chromatography coupled to quadrupole time of flight-MS (LC-QTOF/MS) System (Agilent Technologies 1290 LC, 6540 MS, Agilent Technologies, Santa Clara, CA, USA) using reverse phase (RP) combined with positive ion ESI mode. A Zorbax Eclipse XDBC18 column (100 × 2.1 mm, 1.8 μm; Agilent Technologies) was used at 45°C and flow rate 0.6 mL/min with solvent A—water with 0.1% formic acid, and solvent B—acetonitrile with 0.1% formic acid. The gradient initiated from 25 to 95% B in 35 min, and returned to starting conditions in 1 min, with there-equilibration with 25% B for 9 min. For data acquisition, the TOF mass range was set from 50 to 1000 m/z. During the analysis two reference masses: 121.0509 m/z (C5H4N4) and 922.0098 m/z (C18H18O6N3P3F24) were continuously measured for constant mass correction and thus obtain the accurate mass. The capillary voltage was 3000 V with a scan rate of 1.0 scan per second. The nebulizer gas flow rate was 10.5 L/min.

Metabolite data were normalized using the dry (lyophilized) weight (DW) of the samples. For all experimental conditions, three independent and randomized runs were performed in all metabolomic analysis.

### RNA extraction

A total of 200 mg of grape berry tissue (without seeds) previously grounded in liquid nitrogen was used for total RNA extraction following the protocol by Reid et al. (2006) in combination with purification with RNeasy Plant Mini Kit (Qiagen). After treatment with DNase I (Qiagen), cDNA was synthesized from 1 μg of total RNA using Omniscript Reverse Transcription Kit of Qiagen.

### Transcriptional analysis by real-time qPCR

The expression of several target genes (Supplementary Table 1) in berries at different developmental stages from control and kaolin treated vines was analyzed by real-time qPCR. For that purpose, cDNA was synthesized from 1 μg of total RNA using Omniscript Reverse Transcription Kit (Qiagen). Real-time PCR analysis was performed with QuantiTect SYBR Green PCR Kit (Qiagen) using 1 μL cDNA (diluted 1:10 in ultra-pure distilled water) in a final reaction volume of 10 μL per well. As reference genes, *VvACT1* (actin), and *VvGAPDH* (glyceraldehyde-3-phosphate dehydrogenase) were selected, as these genes were proven to be very stable and ideal for qPCR normalization purposes in grapevine (Reid et al., 2006). Gene specific primer pairs used for each target or reference gene are listed on Supplementary Table 1 (Downey et al., 2003; Bogs et al., 2006; Conn et al., 2008; Gomez et al., 2009; Boubakri et al., 2013; Conde et al., 2015). Primers specifically designed for this work were obtained with the aid of QuantPrime (Arvidsson et al., 2008). Melting curve analysis was performed for specific gene amplification confirmation. The expression values were normalized by the average of the expression of the reference genes as described by Pfaffl (2001). For all experimental conditions tested, two independent biological runs with mathematical triplicates were performed.

### Protein extraction

Total protein extraction from grape berry powder was performed as described by Stoop and Pharr (1993) with several modifications. Sample powder was thoroughly mixed with extraction buffer in an approximately 1:1 (v/v) powder: buffer ratio. Protein extraction buffer contained 50 mM Tris-HCl pH 8.9, 5 mM MgCl_2_, 1 mM EDTA, 1 mM phenylmethylsulfonyl fluoride (PMSF), 5 mM dithiothreitol (DTT), and 0.1% (v/v) Triton X-100. The homogenates were thoroughly mixed and centrifuged at 18000x*g* for 20 min and the supernatants were maintained on ice and used for all enzymatic assays. Total protein concentrations of the extracts were determined by the method of Bradford (Bradford, 1976) using bovine serum albumin as a standard.

### Phenylalanine ammonia lyase (PAL) enzymatic assay

PAL biochemical activity was determined in crude enzymatic extracts following the trans-cinnamic acid production at 41°C, in a total volume of 2 mL. The reaction mixture contained 0.2 mL of enzyme extract, 3.6 mM NaCl, and 25 mM L-phenylalanine (a saturating concentration that ensured that the reaction occurred at the *V*_max_) as substrate in 50 mM Tris-HCl pH 8.9. The rate of conversion of L-phenylalanine to cinnamic acid was monitored continuously in the spectrophotometer at 290 nm. Reactions were initiated by the addition of L-phenylalanine.

### Flavonol synthase (FLS) enzymatic assay

FLS biochemical activity determination was performed as described by Li et al. (2012) with some modifications. Enzyme extraction was performed as described above, but for FLS activity measurements the extracts were additionally purified with Amicon Ultra 4 Centrifugal Filters (Merck Millipore). FLS activity was determined following quercetin production at 37°C during 1 h in a total volume of 1 mL. The reactions were performed at pH 5.0 with 111 mM sodium acetate, 83 μM 2-oxoglutaric acid, 42 μM ferrous sulfate, 120 μL of enzyme extract and started with 400 μM dihydroquercetin, the substrate, at a saturating concentration that ensured *V*_max_, and the production of quercetin was followed at 365 nm (ε = 13.2 mM^−1^ cm^−1^).

### UDP-glucose:flavonoid 3-O-glucosyltransferase (UFGT) enzymatic assay

The biochemical activity of UFGT was determined as described by Mori et al. (2005), with some adaptations. The assay mixture contained 100 mM sodium phosphate buffer pH 8.0, 1 mM UDP-glucose and 100 μL enzyme extract, in a final volume of 500 μL. The reaction was initiated with 1 mM quercetin as substrate (saturating concentration). The reaction mixture was incubated under gentle shaking for 30 min and the production of quercetin 3-glucoside was followed at 350 nm during 30 min (ε = 21877 M^−1^ cm^−1^).

### Quantification of total phenolics and anthocyanins

The concentration of total phenolics and anthocyanins was performed as described in our previous work (Dinis et al., 2016a). Briefly, the concentration of total phenolics was quantified by the Folin-Ciocalteu colorimetric method in berries from all experimental conditions. Total phenolics were extracted in 1.5 mL of pure methanol from 100 mg of berry grounded tissue. The homogenates were vigorously shaken for 15 min and subsequently centrifuged at 18000x*g* for 20 min. Twenty μL of each supernatant were added to 1.58 mL of deionized water and 100 μL of Folin reagent, vigorously shaken and incubated for 5 min in the dark before adding 300 μL of 2M sodium carbonate. After 2 h of incubation in the dark, the absorbance of the samples was measured at 765 nm. Total phenolic concentrations were determined using a gallic acid calibration curve and represented as gallic acid equivalents (GAE). Anthocyanins were extracted from 150 mg of grape berry grounded tissue with 1 mL of 100% acetone. The suspension was vigorously shaken for 30 min. The homogenates were centrifuged for 20 min at 18000x*g* and the supernatants were collected. Anthocyanin extracts were diluted 1:10 in 25 mM potassium chloride solution pH 1.0 and absorbance was measured at 520 nm and 700 nm, using 25 mM potassium chloride solution pH 1.0 as blank. Total anthocyanin quantification was calculated in relation to cyanidin-3-glucoside equivalents, calculated by equation 1, per DW:

(1) $$\begin{array}{r} \left[Total\;anthocyanins\right]\left(mg∕L\right)=\\ \frac{\left(A_{520}-A_{700}\right)\times MW\times DF\times 1000}{\varepsilon \times 1} \end{array}$$

where *MW* is the molecular weight of cyanidin-3-glucoside (449.2 g mol^−1^), *DF* is the dilution factor and ε is the molar extinction coefficient of cyanidin-3-glucoside (26900 M^−1^ cm^−1^).

### Statistical analysis

The results were statistically analyzed by Student's *t*-test using Prism vs. 5 (GraphPad Software, Inc.). For each condition, statistical differences between mean values are marked with asterisks (^*^P < 0.05; ^**^P < 0.01; ^***^P < 0.001).

## Results

### Effect of exogenous kaolin application on grape berry secondary metabolites

**Figure 1:**
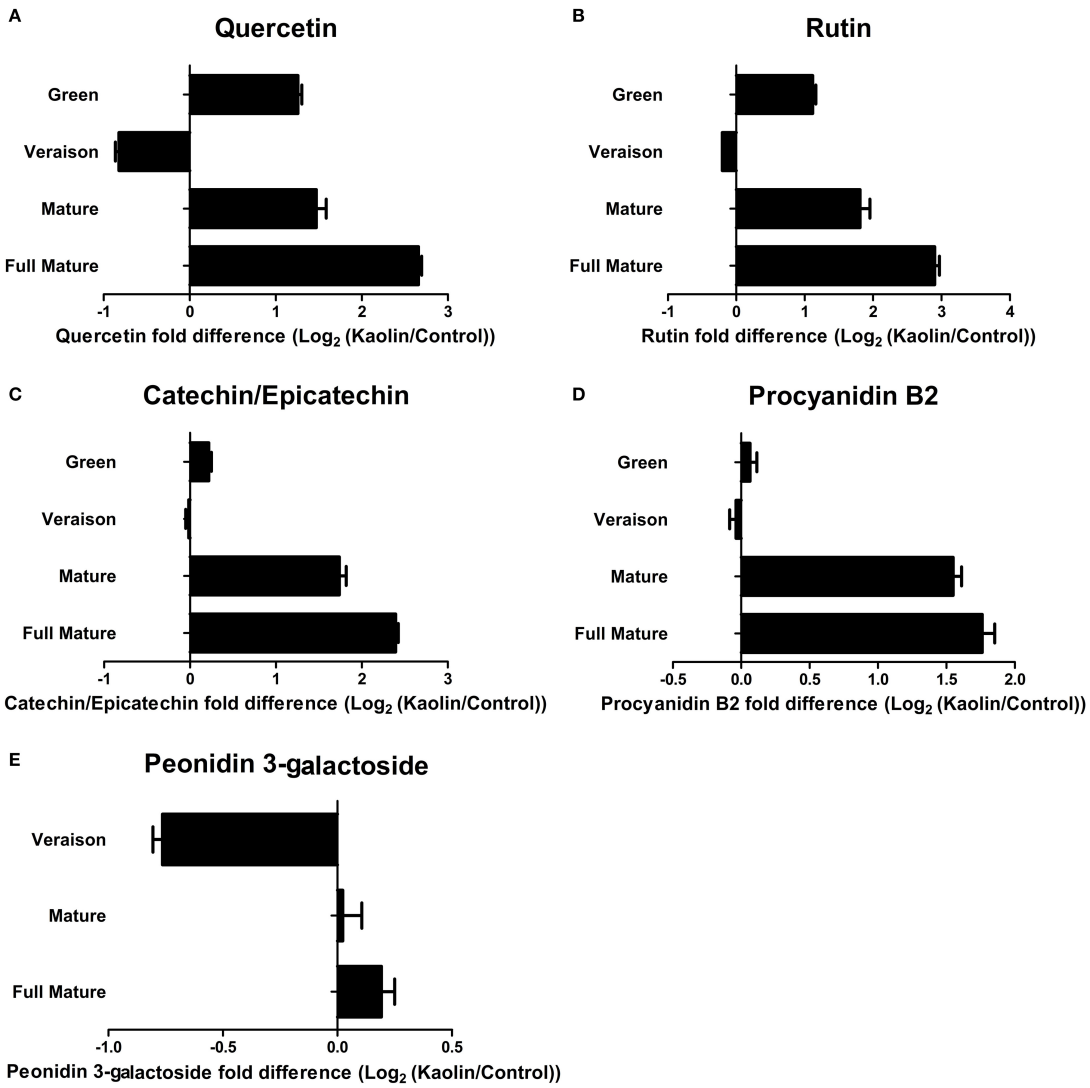
**Effect of kaolin application on the quantities of important secondary metabolites of grape berries**. Relative amounts of **(A)** quercetin; **(B)** rutin; **(C)** catechin/epichatechin; **(D)** procyanidin B2, and **(E)** peonidin 3-galactoside (log2 transformation of kaolin/control fold variation), obtained by reverse phase LC-QTOF-MS, in grape berry tissues collected in four different maturation stages (green, *véraison*, mature, and full mature) from vines subjected to kaolin treatment and without application (control).

In a very recent study (Dinis et al., 2016a) we observed that a foliar treatment of cv. Touriga Nacional with kaolin significantly increased the total amount of phenolic compounds in mature and fully mature berries, and of anthocyanins in fully-mature berries only. Results obtained in the present work confirmed those observations, and demonstrated a significant increase of total phenolics in roughly 30% in the late-green phase, and unchanged concentrations in véraison (Supplementary Figure 1). In agreement, the quantities of quercetin (flavonol), rutin (flavonol glucoside), catechin/epicatechin (monomeric flavan-3-ol), procyanidin B2 (proanthocyanidin), and peonidin 3-galactoside (anthocyanin), which were identified by reverse phase LC-QTOF-MS, were all substantially increased in berries from grapevines treated with kaolin (Figure 1).

**Figure 2:**
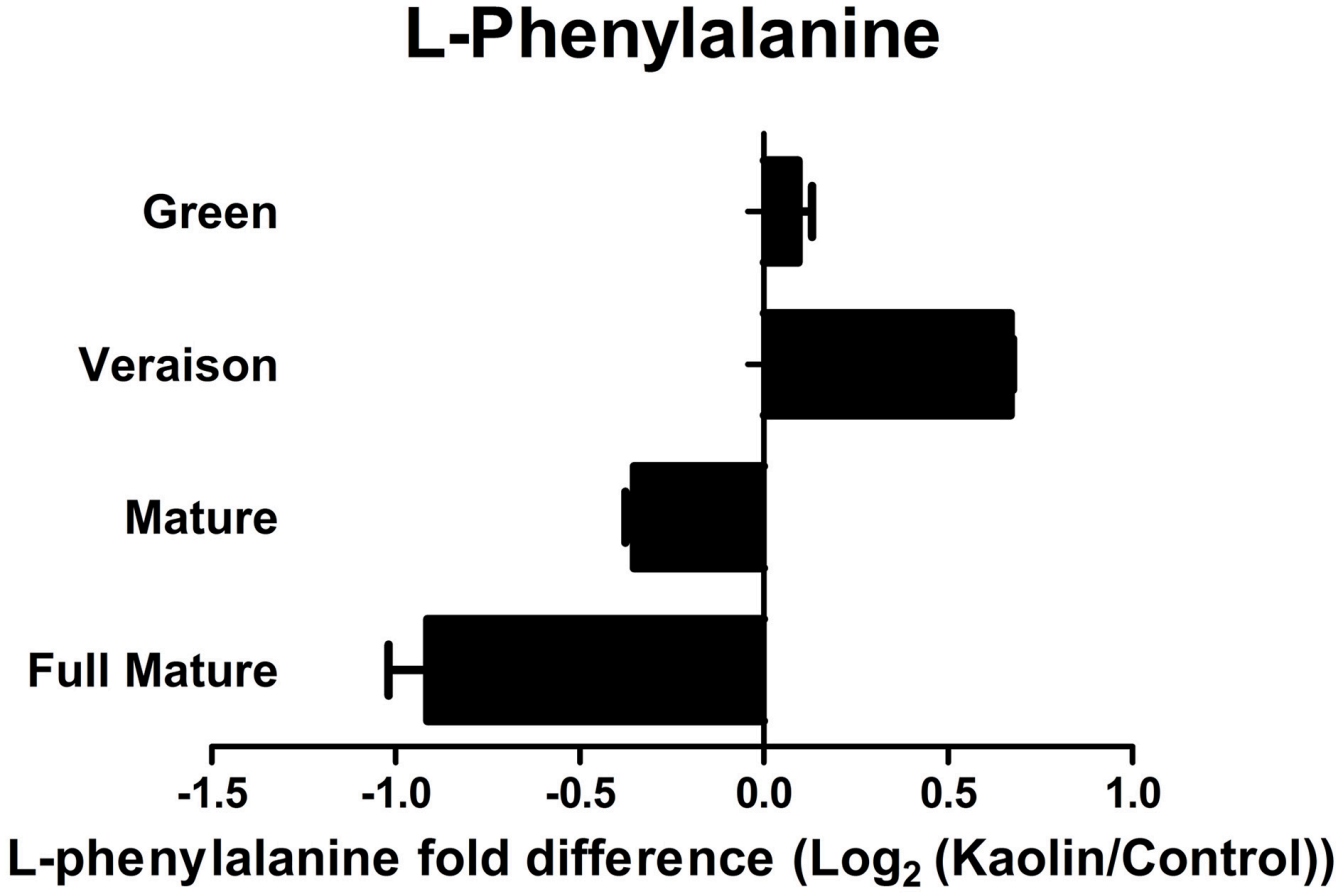
**Effect of kaolin application on L-phenylalanine quantities in grape berries**. Relative amounts of L-phenylalanine (log2 transformation of kaolin/control fold variation) obtained by reverse phase LC-QTOF-MS, in grape berry tissues collected in four different maturation stages (green, *véraison*, mature, and full mature) from vines subjected to kaolin treatment and without application (control).

Noticeably, LC-QTOF-MS analysis also showed that mature and fully mature berries from vines treated with kaolin had a significantly lower quantity of L-phenylalanine, the first metabolite to be converted (into *trans*-cinnamic acid) in the phenylpropanoid pathway, than berries from the control vines (Figure 2).

### Transcriptional and biochemical activity differences in the phenylpropanoid pathway

**Figure 3:**
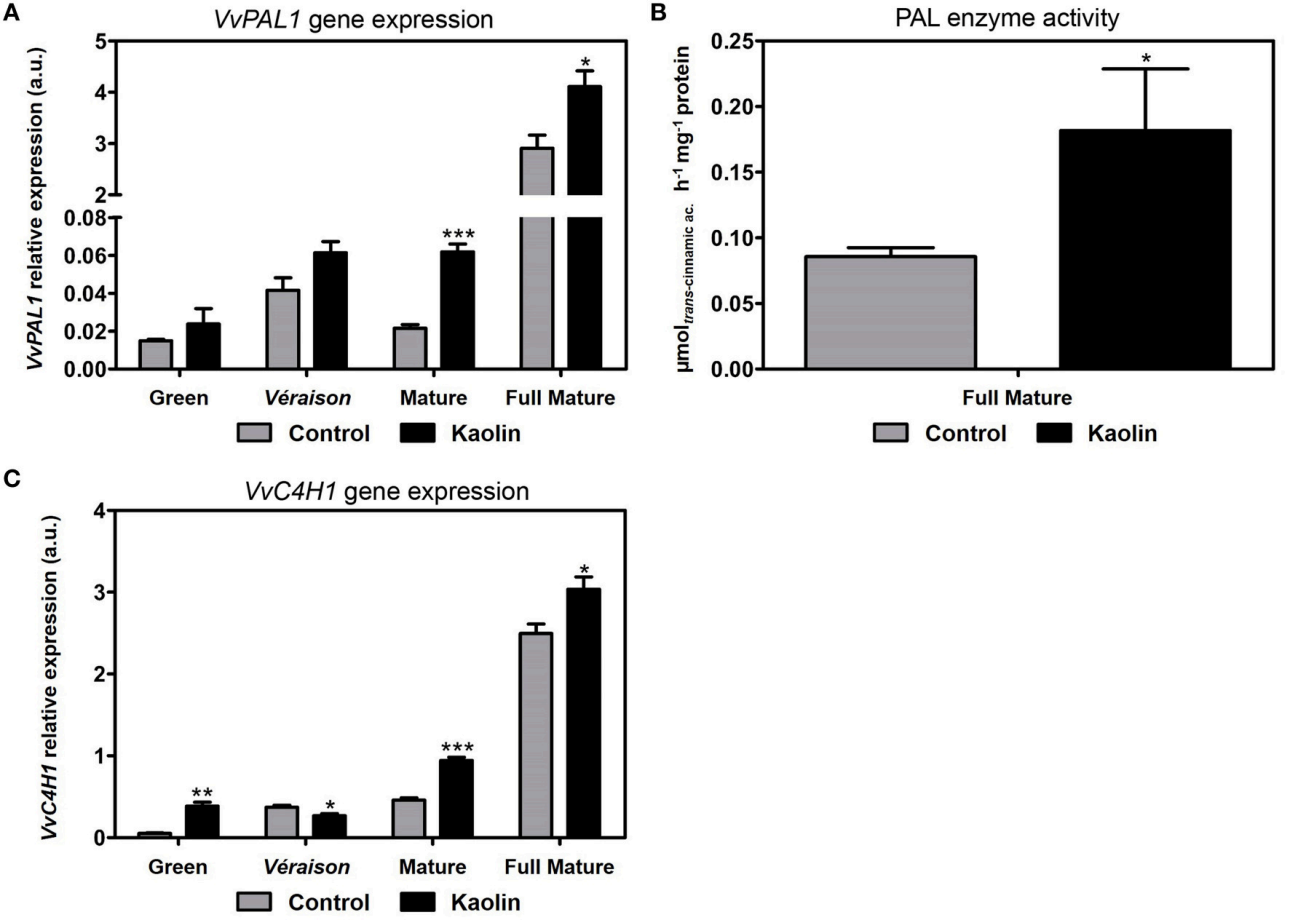
**Stimulatory effect of kaolin on the phenylpropanoid pathway. (A)** Transcript levels of grapevine phenylalanine ammonia lyase 1 (*VvPAL1*) and cinnamate-4-hydroxylase 1 (*VvC4H1*) **(C)** in grape berries. **(B)** Phenylalanine ammonia lyase (PAL) total enzymatic activity, determined as *V*_max_ in berries collected in the full mature stage (12th September) from vines treated with kaolin and without application (control). The assay was performed in triplicate. Values are the mean ± SEM of three independent experiments. Asterisks indicate statistical significance (Student's *t*-test; ^*^P < 0.05). Gene expression analyses were performed by real-time qPCR in grape berry tissues collected in four different maturation stages (green, *véraison*, mature, and full mature) from vines subjected to kaolin treatment and without application (control). *VvPAL1* and *VvC4H1* relative expression levels were obtained after normalization with the expression of the reference genes *VvACT1* and *VvGAPDH*. Two independent PCR runs with triplicates were performed for each tested mRNA. Values are the mean ± SEM. Asterisks indicate statistical significance (Student's *t*-test; ^*^P < 0.05; ^***^P < 0.001).

In our previous report (Dinis et al., 2016a) we showed that the transcript levels of a phenylalanine ammonia lyase gene (*VvPAL1*), that encodes an enzyme catalyzing the first step in the phenylpropanoid pathway in which trans-cinnamic acid is produced, increased in the final maturation stages by 33% in berries from kaolin-treated plants. Here, we observed that *VvPAL1* transcripts also appeared to be slightly more abundant in berries from kaolin treated plants at the late-green and véraison stages (Figure 3A), in a trend that continued until the final maturation phase.

As the highest *VvPAL1* expression level occurred at the final ripening stage, here we determined the total PAL biochemical activity in crude extracts from fully mature berries. In agreement with the increase in *VvPAL1* transcript abundance, results showed a two-fold higher PAL specific activity in berries from vines subjected to kaolin treatment than in control berries (Figure 3B). As shown in Figure 3C, the steady-state transcript abundance of *VvC4H1*, which codes for a cinnamate-4-hydroxylase (C4H) that catalyzes the second reaction in the phenylpropanoid pathway, were also increased in berries from kaolin-treated plants, by 100% and approximately 20% at the mature and full mature stages, respectively.

### Transcriptional changes in stilbene biosynthetic pathway

**Figure 4:**
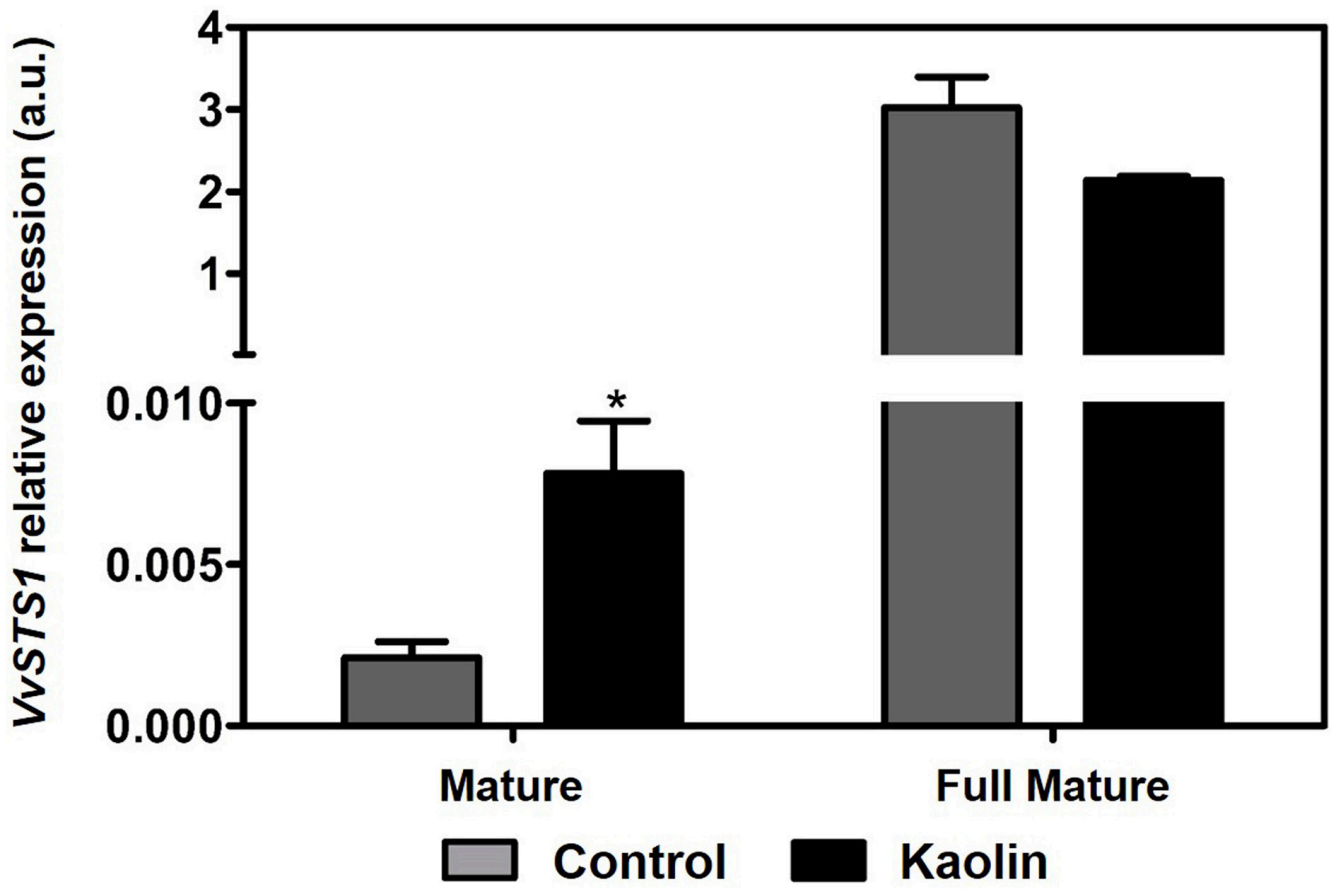
**Effect of kaolin application in the transcript levels of grapevine stilbene synthase 1 (*VvSTS1*) in mature and full mature grape berries**. Gene expression analysis was performed by real-time qPCR in grape berry tissues collected from vines subjected to kaolin treatment and without application (control). *VvSTS1* relative expression levels were obtained after normalization with the expression of the reference genes *VvACT1* and *VvGAPDH*. Two independent PCR runs with triplicates were performed for each tested mRNA. Values are the mean ± SEM. Asterisks indicate statistical significance (Student's *t*-test; ^*^P < 0.05.

To evaluate how the stilbenoid pathway was influenced by foliar kaolin application, transcriptional analysis of *stilbene synthase 1* (*VvSTS1*), that encodes the first enzyme of this pathway, was performed. However, with the primer pair used for amplification, we actually amplified several STS family genes, which can provide a broader sense of the changes in this metabolic pathway. Stilbene synthase (STS) is responsible for the condensation of 4-coumaroyl-CoA with 3 molecules of malonyl-CoA producing resveratrol. The real-time qPCR analysis revealed that *VvSTS* transcript levels increase up to 1000-fold from mature to full mature stages, but kaolin application appeared to stimulate *VvSTS* transcription only in the mature stage (Figure 4).

### Transcriptional and biochemical activity changes in the flavonoid pathway—biosynthesis of flavonols, flavanols, and anthocyanins

**Figure 5:**
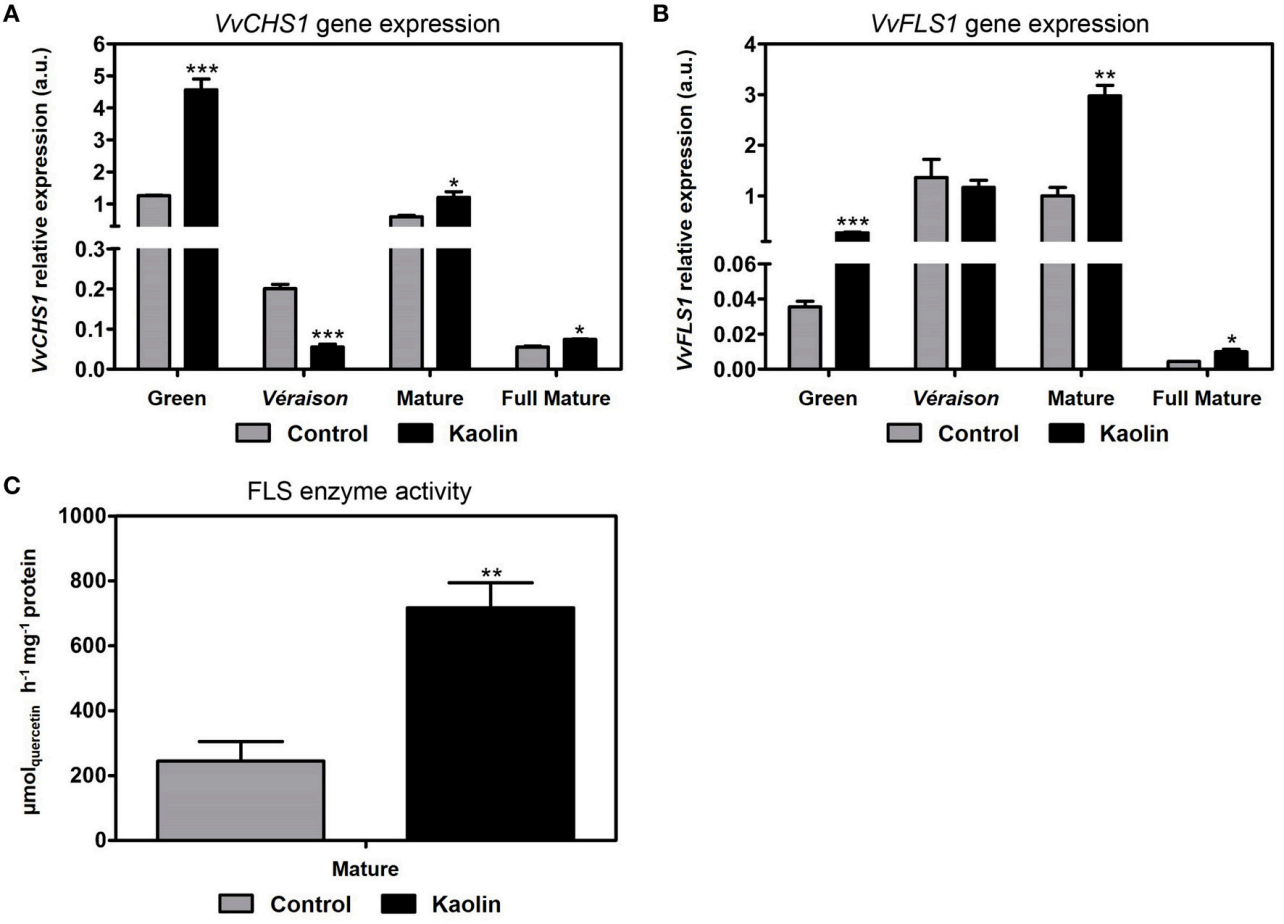
**Stimulatory effect of kaolin on chalcone synthase and flavonol synthase. (A)** Transcript levels of chalcone synthase 1 (*VvCHS1*) and flavonol synthase 1 (*VvFLS1*) **(B)** in grape berries. Gene expression analyses were performed by real-time qPCR in grape berry tissues collected in four different maturation stages (green, *véraison*, mature, and full mature) from vines subjected to kaolin treatment and without application (control). *VvCHS1* and *VvFLS1* relative expression levels were obtained after normalization with the expression of the reference genes *VvACT1* and *VvGAPDH*. Two independent PCR runs with triplicates were performed for each tested mRNA. Values are the mean ± SEM. Asterisks indicate statistical significance (Student's *t*-test; ^*^P < 0.05; ^***^P < 0.001). **(C)** Flavonol synthase (FLS) total biochemical activity, determined as *V*_max_ in berries collected in the mature stage (3rd September) from vines treated with kaolin and without application (control). The assay was performed in triplicate. Values are the mean ± SEM of three independent experiments. Asterisks indicate statistical significance (Student's *t*-test; ^**^P < 0.01).

Transcriptional changes in several important intermediates in the flavonoid pathway were also analyzed. This pathway is initiated by the action of chalcone synthase (CHS). As shown in Figure 5A, the expression of a paradigmatic chalcone synthase gene, *VvCHS1*, which is the better characterized chalcone synthase isoform in grapevine, was not constant during the season and was variably affected by kaolin. The highest steady-state transcript abundance quantity of *VvCHS1* was observed at the late-green stage, when the stimulatory effect of kaolin was more evident (five-fold increase over the control). However, kaolin application also stimulated *VvCHS1* transcription at the mature and full mature stage in a very subtle way, as we had reported before (Dinis et al., 2016a).

#### Flavonol biosynthesis

Flavonol synthase (FLS) is the first enzyme of the flavonol biosynthetic branch of the flavonoid pathway. Gene expression analysis by qPCR revealed that *VvFLS1* was mostly expressed at the *véraison* and mature stages (Figure 5B), and then the steady-state transcript levels decreased abruptly at full mature stage. The stimulatory effect of kaolin application on *VvFLS1* expression was more evident in late green berries and at the mature stage, when a three-fold increase over the control was observed. Concordantly, in berries from kaolin-treated vines, the biochemical activity of FLS was also three-fold higher than in berries from untreated plants (Figure 5C)

#### Flavanol and anthocyanin biosynthesis

**Figure 6:**
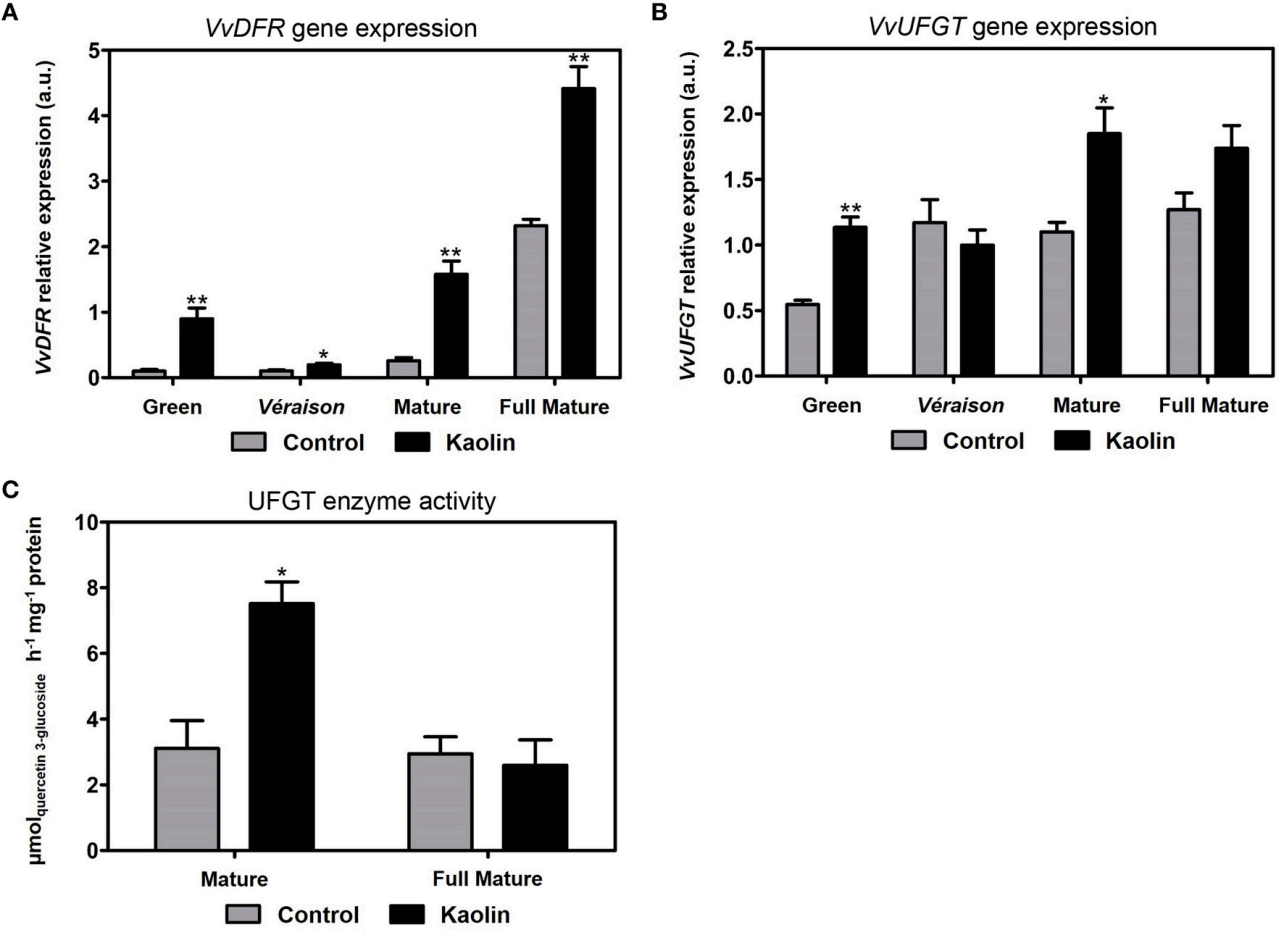
**Stimulatory effect of kaolin on key intervenients of anthocyanin biosynthesis. (A)** Transcript levels of dihydroflavonol reductase (*VvDFR*) and UDP-glucose:flavonol 3-*O*-glucosyl transferase (*VvUFGT*) **(B)** in grape berries. Gene expression analyses were performed by real-time qPCR in grape berry tissues collected in four different maturation stages (green, *véraison*, mature, and full mature) from vines subjected to kaolin treatment and without application (control). *VvDFR* and *VvUFGT* relative expression levels were obtained after normalization with the expression of the reference genes *VvACT1* and *VvGAPDH*. Two independent PCR runs with triplicates were performed for each tested mRNA. Values are the mean ± SEM. Asterisks indicate statistical significance (Student's *t*-test; ^*^P < 0.05; ^**^P < 0.01). **(C)** UDP-glucose:flavonol 3-*O*-glucosyl transferase (UFGT) total biochemical activity, determined as *V*_max_ in berries collected in the mature (3rd September) and full mature (12th September) stages from vines treated with kaolin and without application (control). The assay was performed in triplicate. Values are the mean ± SEM of three independent experiments. Asterisks indicate statistical significance (Student's *t*-test; ^*^P < 0.05).

Dihydroflavonols are secondary metabolites that can enter in either anthocyanin or flavan-3-ol biosynthetic pathways. By catalyzing the reduction of dihydroflavonols to flavan-3,4-diols, the enzyme dihydroflavonol reductase (DFR) is responsible for the first committed step in the pathway leading to the synthesis of flavan-3-ols (or flavanol) compounds, a group that comprises catechin, epicatechin, epigallocatechin, other tannins and proanthocyanidins; and also in the pathway that culminates with the synthesis of anthocyanins. We observed that, in all developmental stages, *VvDFR1* expression was significantly higher in berries from vines treated with kaolin (Figure 6A), with increases by almost six-fold and two-fold, for instance, in the mature and full mature stages. The enzyme UDP-glucose:flavonol 3-*O*-glucosyl transferase (UFGT) catalyzes the final step of anthocyanin biosynthesis. The transcript levels of *VvUFGT* were noticeably higher in kaolin-treated than in control berries, particularly in the green (two-fold) and the mature (80%) stages, but also slightly, yet not statistically significant, in fully mature berries (Figure 6B). In agreement with the transcript abundance of the gene *VvUFGT1*, the UFGT specific activity was significantly enhanced by little more than two-fold, in mature berries from kaolin-treated plants, while no differences were observed in full-mature berries (Figure 6C).

**Figure 7:**
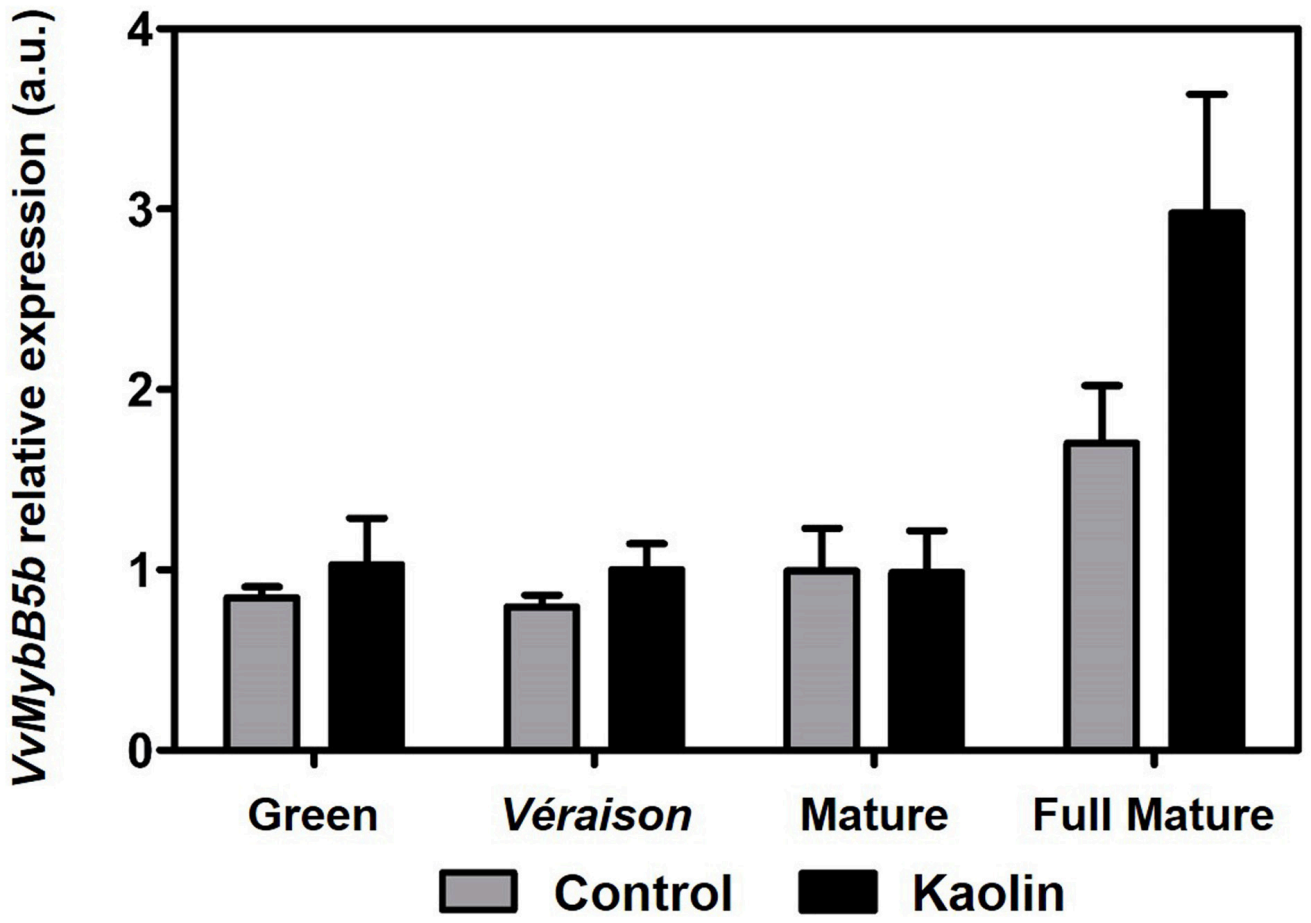
**Effect of kaolin application in the transcript levels of grapevine R2R3-MYB transcription factor family member MybB5b (*VvMybB5b*) in grape berries**. Gene expression analysis was performed by real-time qPCR in grape berry tissues collected in four different maturation stages (green, *véraison*, mature, and full mature) from vines subjected to kaolin treatment and without application (control). *VvMybB5b* relative expression levels were obtained after normalization with the expression of the reference genes *VvACT1* and *VvGAPDH*. Two independent PCR runs with triplicates were performed for each tested mRNA. Values are the mean ± SEM.

The gene *VvMYB5b* encodes a protein belonging to the R2R3-MYB family of transcription factors that has been unequivocally characterized as a regulator of the flavonoid pathway and as having a great role in anthocyanin- and proanthocyanidin-derived compounds accumulation (Deluc et al., 2008). Moreover, it is predominantly expressed during grape berry ripening, making it an ideal candidate to evaluate MYB-related regulation of anthocyanin biosynthetic pathway in the present work. As denoted in Figure 7, *VvMYB5b* appeared to be slightly up-regulated at the full mature stage, when kaolin application seemed to increase its expression, however the differences were not statistically significant between treatments.

### Transcriptional changes in anthocyanin S-conjugation and vacuolar transport

**Figure 8:**
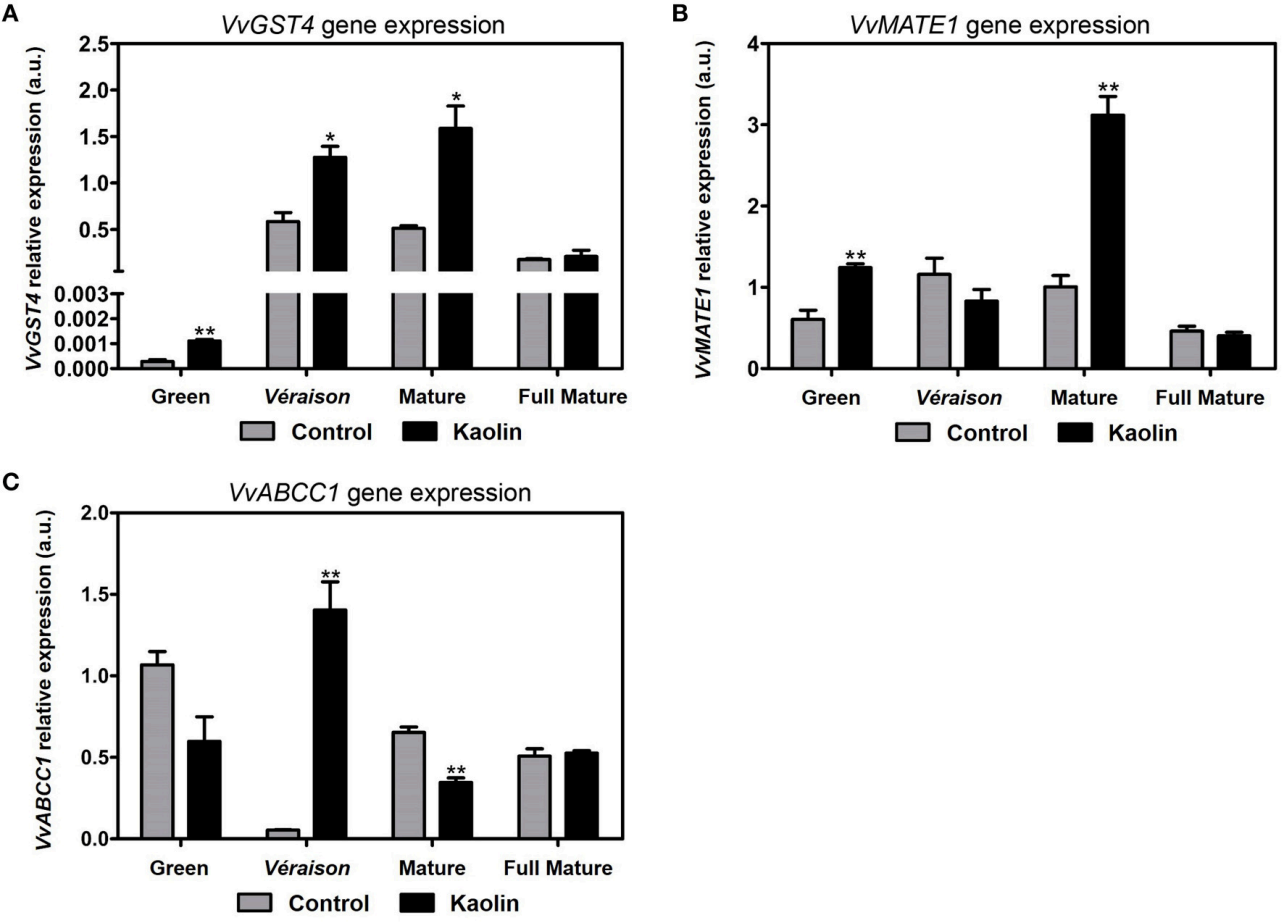
**Stimulatory effect of kaolin on the transcription of genes involved in anthocyanin S-conjugation and vacuolar transport capacity. (A)** Transcript levels of glutathione S-transferase 4 (*VvGST4*) and of anthocyanin tonoplast transporters MATE1 (*VvMATE1*) **(B)** and ABCC1 **(C)** in grape berries. Gene expression analyses were performed by real-time qPCR in grape berry tissues collected in four different maturation stages (green, *véraison*, mature, and full mature) from vines subjected to kaolin treatment and without application (control). *VvGST4, VvMATE1*, and *VvABCC1* relative expression levels were obtained after normalization with the expression of the reference genes *VvACT1* and *VvGAPDH*. Two independent PCR runs with triplicates were performed for each tested mRNA. Values are the mean ± SEM. Asterisks indicate statistical significance (Student's *t*-test; ^*^P < 0.05; ^**^P < 0.01).

Transcriptional changes in genes involved in anthocyanin S-conjugation and in vacuolar transport for intracellular storage were also evaluated. The expression of the gene *VvGST4*, coding for glutathione S-transferase 4, was higher in berries under kaolin treatment in all development stages except in the full mature, with the three-fold increase in mature berries being most noticeable (Figure 8A). This enzyme is key in stabilizing anthocyanins by conjugating them with the reduced form of glutathione (GSH), a biochemical step that is required for the majority of anthocyanin vacuolar transport (Conn et al., 2008).

Gene expression of the tonoplast anthocyanin transporter *VvMATE1* was also strongly enhanced (by three-fold) in mature berries from kaolin-treated vines, and approximately two-fold higher than the control in the green stage (Figure 8B). On the other hand, the expression of another tonoplast anthocyanin transporter, *VvABCC1*, this one shown to strictly transport S-conjugated anthocyanins only, was very strongly upregulated in kaolin-treated berries at *véraison* by approximately 26-fold (Figure 8C). Interestingly, at the mature stage, the ripening phase when *VvMATE1* expression was very strongly upregulated in response to kaolin, *VvABCC1* transcript levels were higher in berries from untreated plants.

## Discussion

This work, as well as a previous one (Dinis et al., 2016a) reinforce that the treatment of grapevine leaves with the inert clay mineral kaolin increases, in the mature grape berry, the quantities of phenolic compounds, including total phenolics and anthocyanins. This fact should have major implications in fruit and wine quality, while protecting plant against abiotic stress. Here, an analysis focused on secondary metabolism by reverse phase LC-QTOF-MS confirmed that the production in the grape berry of different classes of phenolic compounds—including flavonols, flavonol glucosides, flavan-3-ols, proanthocyanidins and anthocyanins, was indeed generally stimulated by foliar kaolin treatment of Touriga Nacional grapes. Furthermore, we showed here that the higher phenolic/anthocyanin content in response to kaolin is clearly due to a global stimulation of phenylpropanoid, flavonoid—flavonol and anthocyanin—pathways at the gene expression and/or protein activity (enzyme activity) levels. Indeed, a concerted and general increased in the expression of many genes involved in these pathways, along with a significant increase in measured enzymatic activities were observed in the latter ripening stages.

Both *VvPAL1* and *VvC4H1* had higher expression in mature and fully mature berries from kaolin-treated vines, confirming that kaolin enhances this particular pathway that is fundamental for the following synthesis of stilbenes and flavonoids. The observed higher PAL enzymatic activity in fully mature berries from kaolin-treated vines also corroborates this assumption, and suggests that in this case the increased transcription levels of one PAL isoform (*VvPAL1*) do indeed provide strong evidence of a final increased biochemical activity. This increased biochemical activity of PAL, that is the result from the joint activity of all isoenzymes, may account for the observed lower levels of L-phenylalanine content in berries from kaolin-treated vines. Dai et al. (2014) demonstrated that increased L-phenylalanine amounts, the main precursor of phenolic biosynthesis, were not correlated with anthocyanin improvement. Here, we were able to observe the same, as lower L-phenylalanine contents were paralleled by an increase in total phenolics in kaolin-treated berries, and in PAL activity, thus, L-phenylalanine consumption.

The flavonols quercetin and rutin (a glycosylated quercetin-derivative) were successfully identified in the metabolomic analysis by LC-QTOF-MS and both were more abundant in berries from kaolin-treated vines especially at the latter ripening stages. This is in agreement with the enhanced flavonol biosynthetic pathway observed in berries from kaolin-treated vines, in particular at the mature stage. At that point, *VvFLS1* expression level was significantly higher in berries from kaolin-treated vines, which correlated very well with a significantly higher FLS activity, in the same proportion. Together with the correlation of PAL activity and *VvPAL1* transcripts, this shows that, in the secondary metabolic pathways we assessed, increased expression levels of a gene can be predictive/indicative of increased final enzymatic activity resulting from all possible isoforms, attesting our prospective qPCR analysis as a robust approach to assess the influence of kaolin on molecular mechanisms/biochemical pathways related with berry quality.

The four-fold increase in *VvCHS1* expression in green berries from kaolin-treated plants, the stage in which its expression was the highest, also suggests that an enhancement of this metabolic step that begins the flavonoids pathway could have played a role in the higher phenolics concentration observed in this phase.

Anthocyanins are responsible for berry color being, thus, an important quality trait of both fruit and red wine production. At the mature stage, berries are actively synthesizing anthocyanins in a process that stagnates in the very final ripening stage when the berries are ready for harvest. Fully mature berries from kaolin-treated vines had significantly more anthocyanins, in a process that appeared to be initiated in the mature phase. This difference could be explained by higher expression of genes involved in anthocyanin biosynthesis and accumulation in the latter ripening stages of berries from kaolin-treated vines. *VvUFGT*, that glycosylates anthocyanidins/flavonols into anthocyanins using UDP-glucose as co-substrate, was indeed more expressed in mature berries from kaolin-treated vines, with a very good correlation with increased total UFGT higher enzymatic activity, just like the case of PAL and FLS, suggesting the increase in the transcription and activity of UFGT was key for increased anthocyanin concentrations. Like in the case of PAL, the enzymatic activity of UFGT is the clear-cut result from the joint action of all UFGT isoenzymes. Upstream, *VvDFR* expression was also enhanced in berries from kaolin-treated vines at the latter ripening stages, suggesting a whole stimulation of the anthocyanin synthesis pathway. Catechins/epicatechins, procyanidin B2, a proanthocyanidin, and the anthocyanin peonidin-3-galactoside were all also present in higher amounts in mature and fully mature berries from kaolin treated vines. This is in perfect agreement with the overall stimulation of phenylpropanoid and flavonoid pathways by foliar kaolin application. Moreover, anthocyanin stabilization and transport into the vacuole was also increased in berries during the major color change phases (*véraison* and mature) from kaolin-treated vines as demonstrated by increased *VvGST4, VvMATE1*, and *VvABCC1* transcripts.

Anthocyanin accumulation in the grape berry is known to be impaired by high temperatures (Spayd et al., 2002; Yamane et al., 2006; Mori et al., 2005, 2007), which suggests that the fact that foliar kaolin application leads to lower canopy temperatures might also contribute for the higher anthocyanin concentration in berries from kaolin-treated plants. Low anthocyanin accumulation at high temperatures has been reported to result from down-regulation of genes involved in anthocyanin biosynthesis (Mori et al., 2005, 2007; Carbonell-Bejerano et al., 2013).

Mild water deficit has been observed to increase total anthocyanins and stilbenoids (Deluc et al., 2009, 2011; Castellarin et al., 2007a,b, 2012), and to up-regulate genes involved in the phenylpropoanoid biosynthetic pathway (Deluc et al., 2009; Castellarin et al., 2007a,b, 2012). However, severe water deficit causes the opposite and results in lower anthocyanin synthesis and contents. Our results suggest that foliar kaolin application somehow had a stimulatory effect in phenolic and anthocyanin synthesis capacity, and a possible reduction of a severe water deficit stress to a milder form of stress induced by foliar kaolin application should not be ruled out. The recognized capacity of kaolin particle film in reducing part of the radiation that reaches plant tissues, thereby reducing canopy temperature and alleviating heat stress and sunburn, while stimulating photosynthesis (Dinis et al., 2016b), might also contribute for higher phenolic/anthocyanin concentrations in berries from kaolin-treated plants observed in this study, but a possible direct influence of silicon (Si) should not be ruled out, despite the reported inert nature of kaolin, and future studies to address this matter could provide valuable new insights, following previous reports showing that plants actively respond to Si supplementation, administrated in roots in forms other than kaolin, including the accumulation of phenolics in rice (Zhang et al., 2013) and banana (Fortunato et al., 2014). It is also important to note that kaolin is known for increasing photosynthetic capacity in leaves, therefore increasing the synthesis of photoassimilates like sucrose. Interestingly, gene expression of several sugar transporters with a role in phloem unloading and/or post-phloem loading was increased in mature leaves and, most importantly, in mature and fully-mature berries (not shown) which might indicate an increased sugar transport capacity at the berry level as well as its accumulation or availability for feeding other metabolic pathways. In fact, several studies have shown a relationship between sugar and anthocyanin content (Pirie and Mullins, 1977; Hunter et al., 1991; Larronde et al., 1998; Dai et al., 2014), which suggests that sugar is important for the synthesis of secondary metabolites. Thus, is plausible that kaolin-induced higher sugar transport and availability in the berry might also contribute to the stimulation of these secondary pathways. A somewhat interesting observation appears to be the very few changes generally observed at véraison. Abscisic acid concentration increases to reach its peak at this developmental phase of the berry and is responsible for the beginning of berry coloring and ripening phase initiation (Castellarin et al., 2016), events that are markedly noticed by anthocyanin and other flavonoids accumulation. A possible explanation for the the fact that kaolin had no apparent effect at veraison might very well be the large concentrations of ABA comparing to the other phases, so that the regulation exerted by this hormone heavily controls the expression of the molecular mechanisms behind flavonoid and anthocyanin synthesis and superimposes any possible modification induced by the foliar kaolin treatment.

It is also important to note that, despite the absence of a factual skin:pulp ratio measurement in this study, no apparent changes in that regard were observed when collecting and processing the berry samples. So, despite not possible to completely rule out the influence of a slightly modified skin:pulp ratio by foliar application of kaolin, it appears not to be a contributor to the observed stimulated phenylpropanoid- and flavonoid-associated molecular mechanisms. In addition, no apparent changes were observed in berry softening, and alterations of brix, berry size and weight, and total acidity were negligible. However, a small contribution of possible indirect kaolin-induced skin thickening and/or phenology displacement, even though not apparent in the current work, should not be completely ruled out, and should be carefully evaluated in future studies to confirm whether or not they are partially responsible for our observations on phenylpropanoid and flavonoid pathways. Additionally, thoroughly determined ripening indicators throughout berry development such as pH value or titratable acidity and total sugar content in a statistically significant manner is equally important to confirm that no phenology displacement occurs as consequence of foliar kaolin application.

**Figure 9:**
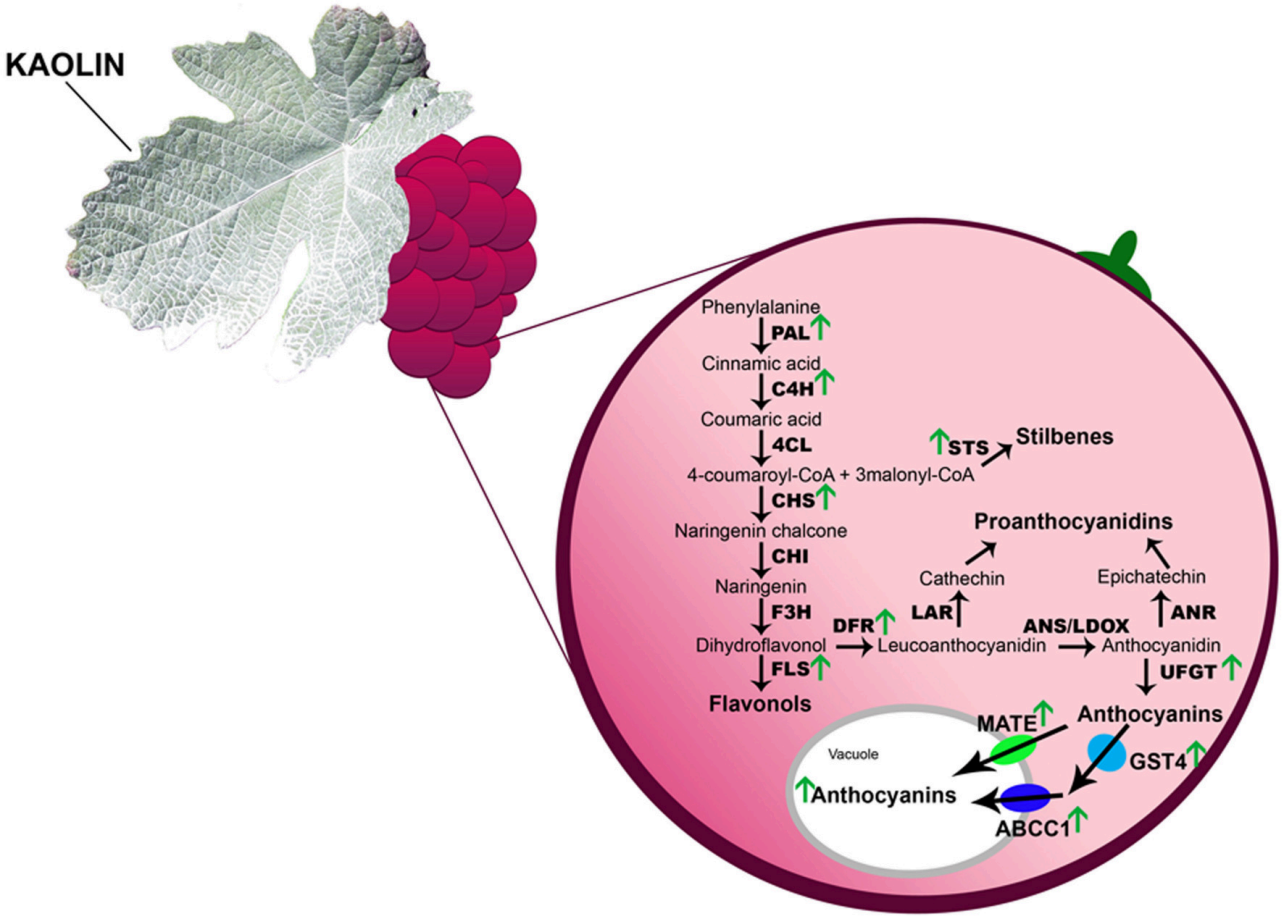
**Foliar kaolin application induced a general stimulation of phenylpropanoid and flavonoid pathways in grape berry cells**. Molecular mechanisms studied in the present work are identified by the upper pointing green arrows that highlight increases of transcripts, biochemical activity or both we observed at any given point during berry ripening in response to kaolin application. CHS, chalcone synthase; CHI, chalcone isomerase; F3H, flavanone 3-hydroxylase; DFR, dihydroflavonol 4-reductase; ANS, anthocyanidin synthase; LDOX, leucoanthocyanin dioxygenase; LAR, leucoanthocyanidin reductase; ANR, anthocyanidin reductase; UFGT, UDPglucose:flavonoid 3-*O*-glucosyltransferase; FLS, flavonol synthase; GST4, glutathione *S*-transferase 4; MATE, anthocyanin multidrug and toxic extrusion transporters; ABCC1, ATP-binding cassette transporter; v, vacuole; cw, cell wall.

In the present work, we showed that grape berries from kaolin-treated vines demonstrated generally enhanced phenolic-biosynthetic molecular mechanisms (Figure 9) that ultimately resulted in higher concentration of phenolics, including anthocyanins. These metabolic pathways are tightly associated with berry quality, and better grape berry quality translates into better wine quality, so, into added value to the winemaking industry, as these compounds are responsible for wine organoleptic properties, like color, flavor, astringency, and bitterness. The conjugation of kaolin application with other mitigation strategies based on viticultural practices or the application of other protective compounds with similar characteristics could also be potentially explored in the future.

In sum, exogenous kaolin application in grapevine leaves shows great potential as summer stress mitigation strategy because it positively impacts berry quality as a result of many molecular and biochemical changes in key secondary metabolic pathways such as phenylpropanoid and flavonoid pathways.

## Author contributions

AC, HG, and JM designed the experiments. AC, DP, AN, LD, SB, and CC performed the experiments. AC and DP analyzed the data. AC, DP, and HG wrote the article. JM directed the study. All authors read and approved the manuscript.

## Funding

The work was supported by European Union Funds (FEDER/COMPETE-Operational Competitiveness Programme—INNOVINE—ref. 311775, Enoexcel—Norte—07-0124- FEDER-000032 and INTERACT - NORTE-01-0145-FEDER-000017 - Linha VitalityWine - ON 0013), and by Portuguese national funds (FCT-Portuguese Foundation for Science and Technology) under the project FCOMP-01-0124-FEDER-022692. AC was supported by Enoexcel—Norte—07-0124-FEDER-000032 and INTERACT - NORTE-01-0145-FEDER- 000017.

### Conflict of interest statement

The authors declare that the research was conducted in the absence of any commercial or financial relationships that could be construed as a potential conflict of interest.
