# Supplementary material for: Kaolin Foliar Application Has a Stimulatory Effect on Phenylpropanoid and Flavonoid Pathways in Grape Berries
Source: Front Plant Sci. 2016 Aug 8;7:1150. doi: 10.3389/fpls.2016.01150 (PMC4976103; doi:10.3389/fpls.2016.01150)
Supplement: Supplementary file 1 [file Table1.PDF]

Supplementary Table 1. Primers forward (F) and reverse (R) used for gene expression analysis by qPCR.

Gene	Accession Number (Genoscope)	Primers	Ref. Primers
VvPAL1	GSVIVG01025703001	F: 5′-CCGAACCGAATCAAGGACTG-3′<br>R: 5′-GTTCCAGCCACTGAGACAAT-3′	Boubakri et al. (2013)
VvC4H1	GSVIVG01024554001	F: 5′-AAAGGGTGGGCAGTTCAGTT-3′<br>R: 5′-GGGGGGTGAAAGGAAGATAT-3′	Boubakri et al. (2013)
VvSTS1*	DO366301[a]	F: 5′-CGAAGCAACTAGGCATGTGT-3′<br>R: 5′-CTCCCCAATCCAATCCTTCA-3′	Boubakri et al. (2013)
VvCHS1	GSVIVT01032968001	F: 5′- GTCCCAGGGTTGATTTCCAA-3′<br>R: 5′- TCTCTTCCTTCAGACCCAGTT-3′	Boubakri et al. (2013)
VvFLS1	GSVIVT01008913001	F: 5’-CAGGGCTTGCAGGTTTTTAG-3’<br>R: 5’-GGGTCTTCTCCTTGTTCACG-3'	Downey et al. (2003)
VvUFGT1	GSVIVT01024419001	F: 5’–TGCAGGGCCTAACTCACTCT–3’<br>R: 5’–GCAGTCGCCTTAGGTAGCAC–3’	Designed with the aid of QuantPrime (Arvidsson et al. 2008)
VvGST4	GSVIVG01035256001	F: 5’-AAGGATCCATGGTGATGAAGGTGTATGGC-3'<br>R: 5’-AACTGCAGAAGCCAACCAACCAACAAAC–3’	Conn et al. (2008)
VvMATE1	GSVIVT01028885001	F: 5’-TGCTTTTGTGATTTTGTTAGAGG-3’<br>R: 5’-CCCTTCCCCGATTGAGAGTA-3’	Gomez et al. (2009)
VvABCC1	GSVIVT01028722001	F: 5’-CTCCACTGGTCCTCTGCTTC-3’<br>R: 5’-AGCCTGCTTCGAAAGTACCA-3’	Designed with the aid of QuantPrime (Arvidsson et al. 2008)
VvACT1	GSVIVT01026580001	F: 5’-GTGCCTGCCATGTATGTTGCC-3’<br>R: 5’-GCAAGGTCAAGACGAAGGATA-3’	Conde et al. (2015)
VvGAPDH	GSVIVT00009717001	F: 5’-CACGGTCAGTGGAAGCATCAT-3’<br>R: 5’-CCTTGTCAGTGAACACACCAG-3’	Conde et al. (2015)
VvDFR	GSVIVT01009743001	F: 5′-GGCTTTCTAGCGAGAGCGTA-3′<br>R: 5′-ACTCTCATTTCCGGCACATT-3′	Bogs et al. (2006)

* The used primers are not very specific and amplify several STS genes including *VvSTS1*

[a] Gen Bank accession number of *VvSTS1*

Arvidsson, S., Kwasniewski, M., Riaño-Pachón, D. M., & Mueller-Roeber, B. (2008). QuantPrime–a flexible tool for reliable high-throughput primer design for quantitative PCR. *BMC bioinformatics*, *9*(1), 1.

Bogs J, Ebadi A, McDavid D, Robinson SP (2006) Identification of the flavonoid hydroxylases from grapevine and their regulation during fruit development. Plant Physiol 140 279–291

Boubakri, H., Poutaraud, A., Wahab, M. A., Clayeux, C., Baltenweck-Guyot, R., Steyer, D., ... & Soustre-Gacougnolle, I. (2013). Thiamine modulates metabolism of the phenylpropanoid pathway leading to enhanced resistance to *Plasmopara viticola* in grapevine. *BMC plant biology, 13*(1), 31.

Conde, A., Regalado, A., Rodrigues, D., Costa, J. M., Blumwald, E., Chaves, M. M., & Gerós, H. (2015). Polyols in grape berry: transport and metabolic adjustments as a physiological strategy for water-deficit stress tolerance in grapevine. *Journal of experimental botany*, *66*(3), 889-906.

Conn, S., Curtin, C., Bezier, A., Franco, C., and Zhang, W. (2008). Purification, molecular cloning, and characterization of glutathione S-transferases (GSTs) from pigmented *Vitis vinifera* L. cell suspension cultures as putative anthocyanin transport proteins. *J. Exp. Bot.* 59, 3621–3634. doi:10.1093/jxb/ern217.

Downey M, Harvey J, Robinson S. Synthesis of flavonols and expression of flavonol synthase genes in the developing grape berries of Shiraz and Chardonnay (*Vitis vinifera* L.) Aust J Grape Wine Res. 2003;9:110–121.

Gomez, C., Terrier, N., Torregrosa, L., Vialet, S., Fournier-Level, A., Verries, C., et al. (2009). Grapevine MATE-Type Proteins Act as Vacuolar H+-Dependent Acylated Anthocyanin Transporters. *PLANT Physiol.* 150, 402–415. doi:10.1104/pp.109.135624.
