# Supplementary material for: Kaolin Foliar Application Has a Stimulatory Effect on Phenylpropanoid and Flavonoid Pathways in Grape Berries
Source: Front Plant Sci. 2016 Aug 8;7:1150. doi: 10.3389/fpls.2016.01150 (PMC4976103; doi:10.3389/fpls.2016.01150)
Supplement: Supplementary file 2 [file Image1.PDF]

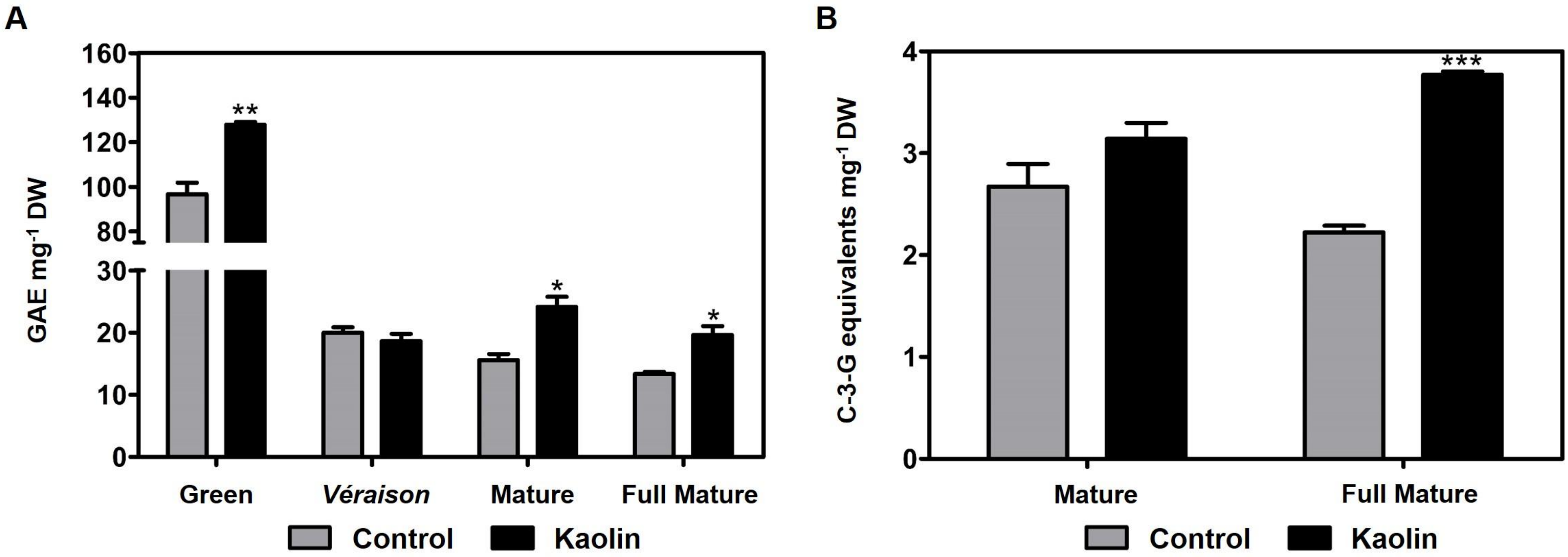

Supplementary Figure 1. Effect of kaolin application on total phenolics (A) and total anthocyanin (B) concentration in grape berries. Total phenolic compounds concentration is represented as µg mL^{-1} of gallic acid equivalents (GAE) per mg of dry weight (DW) and anthocyanin concentration is represented as µg mL^{-1} of cyanidin-3-glucoside (C-3-G) equivalents per mg dry weight (DW) in grape berry tissues collected in four different maturation stages (green, véraison, mature and full mature) from vines subjected to kaolin treatment and without application (control). Asterisks indicate statistical significance (Student's t-test; $*P<0.05$; $**P<0.01$). Phenolic and anthocyanin concentrations in mature and fully mature berries are in agreement with those we had obtained in our previous work (Dinis et al. 2016a)
